# The Functionalization of Activated Polyester Fabrics with Chitosan—Changes in Zeta Potential and Moisture Management

**DOI:** 10.3390/ma17235987

**Published:** 2024-12-06

**Authors:** Ivana Čorak, Anita Tarbuk, Tihana Dekanić, Dominik Sikorski, Zbigniew Draczyński

**Affiliations:** 1 Department of Textile Chemistry and Ecology, University of Zagreb Faculty of Textile Technology, Prilaz baruna Filipovića 28a, HR-10000 Zagreb, Croatia; ivana.corak@ttf.unizg.hr (I.Č.); tihana.dekanic@ttf.unizg.hr (T.D.); 2 Institute of Textile Materials and Polymer Composites, Lodz University of Technology, ul. Żeromskiego 116, 90-924 Lódź, Poland; dominik.sikorski@dokt.p.lodz.pl (D.S.); zbigniew.draczynski@p.lodz.pl (Z.D.)

**Keywords:** polyester fabric, chitosan functionalization, mechanical properties, whiteness, durability, zeta potential, SEM, moisture management

## Abstract

In the interests of using green and sustainable chemical innovations to create sustainable products with minimized (or no) chemical hazard potential, the polyester fabric in this work was activated and functionalized with chitosan and its durability was investigated. Chitosan is a natural biopolymer derived from chitin. As it has good biocompatibility, bio-absorption, anti-infectious, antibacterial and hemostatic properties and accelerates wound healing, it is increasingly being researched for the antimicrobial treatment of textiles. Due to the increased demands on the durability of antimicrobial properties during care, its binding to cellulose in cotton and cotton–polyester blends has been researched, but not to polyester alone. Therefore, the functionalization of polyester fabrics with chitosan by thermosol in the form of submicron particles and pad-dry-curing with homogenized gel was investigated in this work. The functionalization with chitosan was carried out on untreated polyester fabric and polyester fabric activated by alkali hydrolysis. In order to reduce the release of chemical substances during the entire life cycle of textile production, no binder was used. The effects were evaluated by electrokinetic analysis (zeta potential), and the mechanical, spectral, moisture management and antimicrobial properties were determined using standard methods. The functionalized polyester fabrics were submitted to 10 washing cycles in a solution of non-ionic surfactant for determination of its durability. It was shown that the functionalization of hydrolyzed polyester fabric with homogenized chitosan gel by pad-dry-curing results in excellent antimicrobial efficacy and moisture management properties while maintaining the mechanical properties of the fabric even after 10 washing cycles.

## 1. Introduction

Polyester fibers are semi-crystalline materials that have both a crystalline and a non-crystalline part. The crystalline part is usually about 60%, but in highly oriented fibers, the crystallinity can be up to 80%. In addition, the aliphatic-aromatic composition of polyester fibers leads to a low number of active groups, as there are two methylene groups (–CH_2_) in the aliphatic part, which are linked to an aromatic segment by an ester group (–COO–), so that polyester fabrics have high strength but poor water absorption, which leads to poor moisture management and makes the fabrics uncomfortable to wear. It is therefore necessary to modify the fiber surface to increase the number of active groups that promote water absorption and transpiration. This can be achieved by coating, grafting, plasma and UV laser treatment, as well as chemically by hydrolysis with alkalis and enzymes, aminolysis with amines and, more recently, with ionic liquids [1,2,3,4,5,6,7,8,9,10,11,12,13,14,15,16,17,18,19]. Hydrolysis, aminolysis and/or thermal oxidation cleave the polymer chains and increase the number of terminal hydroxyl (-OH) and carboxyl (–COOH) groups, so that the fabrics have better sorption and dyeing properties. Alkali hydrolysis also leads to a better fabric hand (silk-like soft handle), but causes pilling on the surface, which leads to pitting and has a negative effect on the strength of the fabric [1,2,3,4,5,6,7,8,9,10,11,12,13,14]. In addition, a large amount of hot, aqueous alkali solution, usually sodium hydroxide, is required and a lot of energy and water is consumed, as alkali hydrolysis is usually carried out at temperatures above 100 °C for over an hour. The optimum weight loss is between 10 and 24% with a reduction in breaking strength of up to 35%, which is achieved by monitoring alkali concentration, time and temperature [1,6,7,8,9]. The process of alkali hydrolysis can be accelerated by the addition of accelerators—some cationic surfactants and polymers (but not all) [13,19]. Nowadays, quaternary ammonium compounds with at least 16 C atoms such as cetylpyridinium chloride (CPC), hexadecyltrimethylammonium bromide (CTAB) or chloride (HDTMAC), or benzalkonium chloride are used for this purpose [8,9,10,11,12,13,14,19]. Recently, it has been shown that alkali hydrolysis can be carried out at a lower temperature of 80 °C with the addition of HDTMAC as an accelerator, which is more economically and energetically acceptable than the conventional process [13,14]. Therefore, in this study, the surface of the polyester fabric is activated in this more sustainable way.

In the finishing of polyester fabrics, the thermosol process is the most important process for dyeing with disperse dyes and for optical brightening with disperse fluorescent whitening agents (FWAs). It is an anhydrous process based on the fact that diffusion is an activated process, so that the diffusivity of the disperse dye molecules increases with increasing temperature [20]. The polyester substrate is impregnated with the dye, dried and then cured for 60–90 s at very high temperatures, usually 180–220 °C [20,21]. The particle size of disperse dyes and disperse FWAs is very important for the success of dyeing and finishing polyester in the thermosol process. Braun [20] investigated the particle size of disperse dyes and the solubility of such substances in the dispersed state as well as various factors and came to the conclusion that the optimum size of disperse dyes/particles for thermosol is between 0.1 and 1 µm. In addition to dyes and FWA, TiO_2_ and ZnO particles in the submicron range are often used for the thermosol process to reduce gloss due to light scattering and to provide UV protection. New technologies and grinding systems enable the size reduction of solid particles down to submicron scale and even nanoparticles. It is therefore now possible to implement them into polyester fabrics using the thermosol process. Grancarić, Tarbuk et al. [22,23,24] implemented micro- and nanoparticles of activated natural zeolite into modified polyester fabric to achieve antimicrobial and UV protection. In this study, submicron particles of solid chitosan were used.

Chitin and its best-known derivative chitosan are solid polysaccharides that originate from the shells of crustaceans, the cell walls of fungi, and the exoskeletons of insects, and are insoluble in water and most organic solvents. Since chitosan has good biocompatibility, bio-absorption, anti-infectious, antibacterial, anti-fungal, and hemostatic properties and accelerates wound healing, it is increasingly being researched for the antimicrobial treatment of textiles [16,19,25,26,27,28,29,30,31,32,33,34,35,36,37,38,39,40,41,42,43,44,45,46,47,48,49,50,51,52].

There are several mechanisms for the antimicrobial action of chitosan against bacteria and microfungi. The most widely accepted mechanism is electrostatic interactions between the cationic groups of chitosan and negatively charged cell walls. Both types of bacteria are negatively charged, although there are differences in the cell walls of the bacteria; Gram-negative have an additional membrane covered with lipopolysaccharides, while Gram-positive have only a membrane and a thicker layer of peptidoglycan containing negatively charged teichoic acids [41,53,54]. In addition, the chitosan chelates metal ions and inhibits bacterial growth. The zeta potential measurements confirmed that both Gram-positive and Gram-negative bacteria have a negative surface charge, but the charge density is different. Depending on the size of the chitosan molecule, it can disrupt the integrity of the membrane, leading to increased membrane permeability, causing proteins and other intracellular components to leak out and kill the bacteria, or it can form an impermeable layer around the cell if the high molecular weight chitosan molecule is unable to penetrate the cell wall and cell membrane, preventing the transport of important solutes into the cell. In relation to microfungi, chitosan has been shown to cause membrane permeability and leakage of proteinaceous material [41,42,43,44,45,46,47].

As already mentioned, the molecular weight of the chitosan and the degree of acetylation (DDA) influence the dissolution rate [38,39,40,41,42,43,44,45,46,47,48,49,50,51,52]. The smaller the particle size and the molecular weight, the faster and more completely the chitosan can dissolve in the acidic medium. This is because the micronized powders have smaller particle sizes and a larger surface area, the powders are amorphous and the chain length is shorter, so the amino groups can interact with more H^+^ ions at the beginning of dissolution and the water molecules can penetrate more easily when the chitosan chains have better mobility. According to Hassan et al. [45,46,47], chitosan has excellent antimicrobial activity when it is in the form of nanoparticles. This is due to the synergy between a charge on the surface and the ability of the small particles to migrate through the bacterial cell, leading to its death.

The acid activation of chitosan allows it to be applied to textiles in various forms and to obtain antimicrobial properties. Previous studies by the authors [40,41] have confirmed that the DDA of chitosan does not change significantly during degradation in organic acid solutions, e.g., malic acid, formic acid, lactic acid and acetic acid. The degradation leads to the formation of hydroxyl groups at the C1 and C4 positions, and the aldehyde groups characteristic of the degradation of sugars are not present, confirming that only a shortening of the chitosan polymer chains occurs, as shown by intrinsic viscosity and viscometric average molar mass (Mv) results. In terms of degradation activity, malic acid has the greatest destructive effect on the chitosan macromolecules, followed by formic acid and lactic acid, while acetic acid has the least effect on the change in Mv values. The aforementioned acids have a destructive effect on the chitosan macromolecules, but the changes are not as great as with malic acid [40]. Hydrochloric, citric and acetic acid can be used to achieve antimicrobial properties [31,41], but acetic acid results in the best solubility of the chitosan polymer [40].

The application of chitosan to cotton and cotton blends has been researched, and wash durability has been achieved through the use of crosslinking agents [19,31,32,33,34]. In contrast to cotton, polyester has an extremely low number of active groups that are not reactive, and it is difficult to bind the crosslinking agent, resulting in low durability of the treatment. Therefore, in the context of using green and sustainable chemical innovations to create sustainable products with minimized (or no) chemical hazard potential [55], and to reduce the release of chemical substances during the entire life cycle of textile production, the functionalization of polyester fabrics with chitosan using the thermosol process and the pad-dry-cure process without crosslinking agent was investigated.

## 2. Materials and Methods

### 2.1. Materials

A polyester fabric (WFK 30 A) by wfk-Testgewebe GmbH (Brüggen, Germany) of 100% polyester, mass per unit area 170 g/m^2^, yarn linear density 295 dtex, canvas embroidery, was used in this research. This polyester fabric was produced using the description for the standard cotton fabric defined in DIN ISO 2267:2016 [56].

Sodium hydroxide p.a. (NaOH) and acetic acid 99.5% p.a. (CH₃COOH) were purchased from Gram-mol d.o.o. (Zagreb, Croatia); potassium chloride p.a. (KCl) from Kemika (Zagreb, Croatia); hexadecyltrimethylammonium chloride (HDTMAC, 25% aqueous solution) from Merck KGaA (Darmstadt, Germany); and Felosan NOF from CHT-Bezema (Montlingen, Switzerland).

Chitosan donated by Tricomed SA was used in this research. It is an oyster mushroom (*Pleurotus ostreatus*) chitosan purchased from Chibio BioTECH (Qingdao City, China) having a degree of deacetylation of 90% and molecular weight of 150 kDa.

### 2.2. Treatment Procedures

Alkali hydrolysis was performed for the activation of the polyester surface by the batchwise method in stainless steel bowls from Linitest, (Original-Hanau, Hanau, Germany) with LR 1:20, at 80 °C for 30 min in a bath containing 1.5 mol/L NaOH and 2 g/L HDTMAC as an accelerator [13,14]. Hydrolyzed samples were rinsed in hot water for removal of oligomers, and then impregnated in different chitosan baths without further rinsing or neutralization.

For the functionalization of polyester fabrics, submicron particles of chitosan were used. The submicron particles of chitosan were produced by milling in a Planetary Micro Mill PULVERISETTE 7 premium line (FRITSCH GmbH—Milling and Sizing, Weimar, Germany) using ceramic balls with a diameter of 20 mm for 48 min at 900 rpm. After milling, a water suspension of chitosan powder was formed for separation of the fraction of chitosan particles of size less than 1 μm. The chitosan particle diameter achieved, confirmed by dynamic light scattering (DLS) using a Zetasizer (Malvern Panalytical Ltd., Malvern, UK), was within the range of 1 to 0.5 μm.

The submicron particles of chitosan were applied to polyester fabrics by the thermosol process, similar to the disperse dyeing process. Fabrics were impregnated (padded) in water suspension of 3 g/L chitosan on Foulard (Kovinar, Zagreb, Croatia) with WP 100%, dried at 110 °C for 2 min, followed by impregnation in 0.5 mol/L acetic acid with WP 100%, then dried again at 110 °C for 2 min. Thermosoling (thermocondensation, curing) was performed at 190 °C for 40 s. Drying and curing were performed on a Benz continuous dryer.

The chitosan solution was prepared in acetic acid, which was characterized by the best solution stability and biological effect against bacteria, as well as the best solubility of the chitosan polymer [40,41], as follows: 3 g/L chitosan was placed in 0.5 mol/L acetic acid and stirred for 24 h with a magnetic stirrer at room temperature. Viscosity of 1000 cPs (mPa·s) at 20 °C was confirmed using a viscometer (Fungilab, Barcelona, Spain). The prepared homogenized chitosan solution was applied by the pad-dry-cure process. For functionalization, the fabrics were impregnated (padded) in a homogenized solution of chitosan with WP 100%, dried at 110 °C for 2 min and thermocondensed (cured) at 170 °C for 90 s.

All fabrics after functionalization were washed in distilled water and air-dried.

For the purpose of durability determination, 10 washing cycles were performed in a Polycolor (Mathis AG, Oberhasli, Switzerland) finishing and dyeing machine, having LR 1:10 at 60 °C for 30 min. Instead of laundry detergent, the product was washed with 1 g/L Felosan NOF, a low-foaming stain remover and washing agent without APEO.

**Figure 1:**
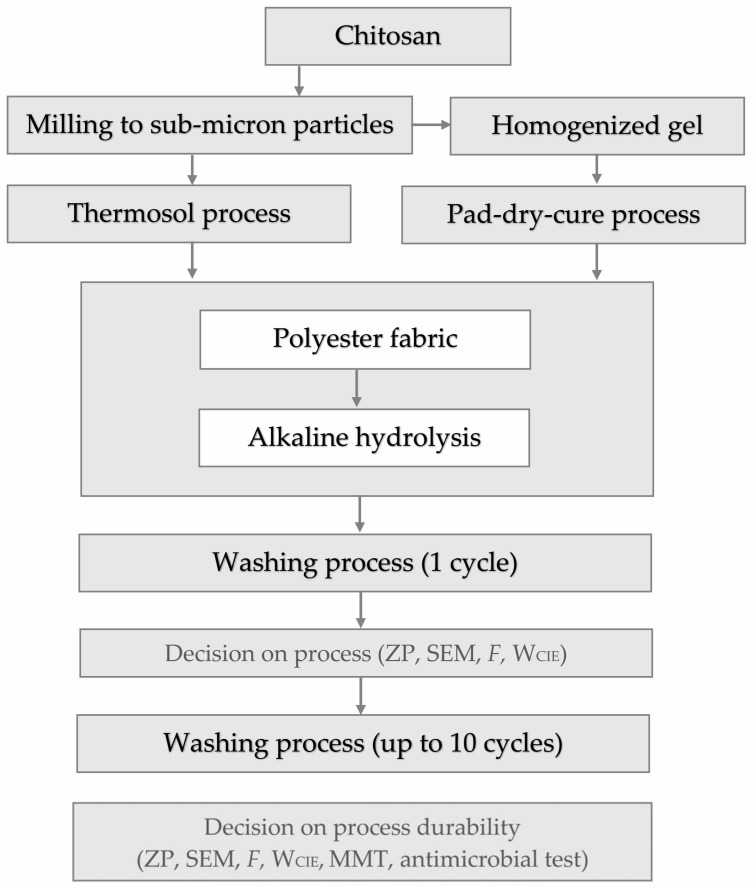
The process flow diagram.

Labels and treatments are listed in Table 1, and the schematic diagram of the process flow is shown in Figure 1.

### 2.3. Characterization Methods

The electrokinetic potential (zeta potential, ZP) was calculated according to the Helmholtz–Smoluchowski equation after measuring the streaming potential with a SurPASS electrokinetic analyzer (Anton Paar GmbH, Graz, Austria) using an Adjustable Gap Cell [57,58]. For each point, the streaming potential was measured 8 times at each pH value, with 2 parallel samples. An electrolyte solution of 0.001 mol/L KCl solution was used for measurement in the pH range from pH 9 to pH 2, and the Isoelectric point (IEP) was determined.

The morphological characterization of the surface of polyester fibers in fabrics was analyzed using photomicrographs taken with a scanning electron microscope (FE-SEM, Mira II, LMU, Tescan, Brno, Czech Republic) with a magnification of 4000×. The samples were coated with a thin layer of chromium for 180 s in a Q150T ES Plus sputter coater (Quorum Technologies, Laughton, UK).

The mass per unit area (*m*) of the fabric in g/m^2^ was determined according to ISO 3801:1977 [59] using an analytical balance with an accuracy of 0.0001 g, model ALJ 220-5DNM (KERN & Sohn GmbH, Balingen, Germany). The sample size was 10 × 10 cm, and there were 10 parallel samples. The change in mass per unit area (Δ*m*) was calculated in relation to the start fabric.

Breaking force (F) in N and elongation (ε) in % were determined according to ISO 13934-1:2013 [60] using a dynamometer Tensolab (Mesdan S.p.A., Puegnago del Garda, Italy). The strip size was 50 mm × 200 mm, clamping distance was 100 mm, pretention was 2 N, and there were 5 parallel samples in warp direction. The changes in breaking force and elongation were calculated in relation to the start fabric.

The spectral remission, R [%] was measured on a spectrophotometer Spectraflash SF 300 (Datacolor, Switzerland). The measurements were performed under standard D_65_ light, using sphere geometry d/8° and the Datacolor ColorTools program and “Measuring until tolerance” command, which means that at least 10 measurements were made, and the results were accepted only if the total color difference between each measurement was less than 0.1. The degree of whiteness according to CIE (W_CIE_) was automatically calculated in accordance to ISO 105-J02:1997 [61], as well as tint deviation from neutral white standard and its coloristic meanings according to [62]. The Yellowing Index (YI) according to DIN 6167:1980-01 [63] was calculated automatically as well.

The moisture management properties were determined according to AATCC TM 195-2017 [64] using a Moisture Management Tester MMT M290 (SDL Atlas). Sample size was 8 cm × 8 cm, with 5 parallel samples.

The antimicrobial activity was determined according to AATCC TM 147-2016 [65]. Activity was determined against the Gram-positive bacterium *Staphylococcus aureus* ATCC 6538 (*S. aureus*), the Gram-negative bacterium *Escherichia coli* ATCC 8739 (*E. coli*), and the microfungus *Candida albicans* ATCC 10231 (*C. albicans*); there were 5 parallel samples.

## 3. Results

**Figure 2:**
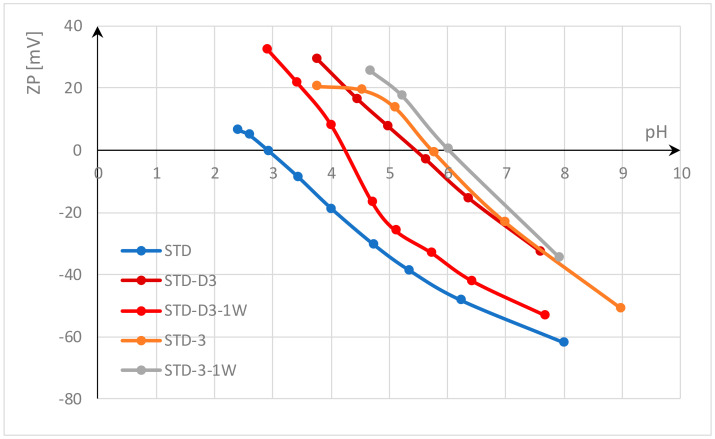
Zeta potential of standard polyester fabrics functionalized with chitosan after treatment and one washing cycle.

**Figure 3:**
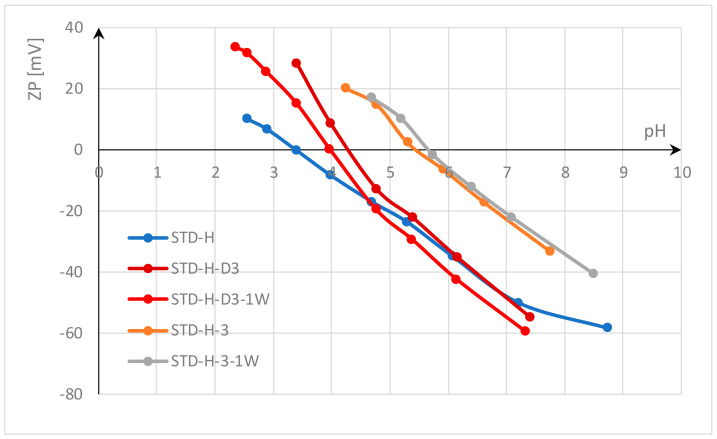
Zeta potential of hydrolyzed polyester fabrics functionalized with chitosan after treatment and one washing cycle.

In this study, the functionalization of polyester fabrics with chitosan was investigated by thermosol in the form of submicron particles and pad-dry-curing with homogenized gel. Functionalization with chitosan was performed on standard polyester fabric and on polyester fabric activated by accelerated alkali hydrolysis with HDTMAC at low temperature. The effects were evaluated by electrokinetic analysis (zeta potential). The results of the zeta potential after functionalization with chitosan and after a wash cycle are shown in Figure 2 for standard polyester fabric and in Figure 3 for alkali hydrolyzed polyester fabric.

The results shown in Figure 2 indicate that the standard (untreated) polyester fabric (STD) has a zeta potential (ZP) of −61.3 mV at pH value 8, and an IEP of 2.95. This negative zeta potential of polyester fabric in an alkaline medium is due to its hydrophobic surface, which results from the low number of active groups due to the high crystallinity of the fibers. In contrast to hydrophilic surfaces, which can absorb water and swell, hydrophobic surfaces have a lower zeta potential because they cannot absorb water molecules [19,57]. Carboxyl and hydroxyl groups modified by alkali hydrolysis can absorb some water and thus increase the ZP of alkali hydrolyzed polyester fabric (STD-H) to −58 mV (Figure 3). This supports the theory of Sanders and Zeronian [2,3] that increased surface roughness, an increase in the number of hydrophilic groups on the fiber surface due to chain scission, and their accessibility lead to the hydrophilic properties of the alkali hydrolyzed polyester fiber. In addition, Latta [4] suggested that the hydroxyl groups are responsible for the hydrophilicity of the hydrolyzed polyester.

Another thing can be observed. Polyester fabrics were activated by alkali hydrolysis accelerated with hexadecyltrimethylammonium chloride (HDTMAC). After hydrolysis, the fabrics were rinsed only once with hot water to remove the oligomers, so there is a high probability that desorption did not take place and some amount of HDTMAC was adsorbed onto the polyester fabric (according to the literature, this may be about 0.01 mmol/g [66]). Even this small amount of cationic surfactant can increase the ZP (ZP at pH 7 for STD is −55.5 mV and for STD-H is −50.1 mV; ZP at pH 6 for STD is −46.7 mV and for STD-H is −35.1 mV; ZP at pH 5 for STD is −35.2 mV and for STD-H is −20.1 mV; etc.) and shift the IEP to a higher pH value; IEP for STD-H is 3.4.

Figure 2 and Figure 3 show that functionalization with chitosan significantly changes the zeta potential of both polyester fabrics. The chitosan hydroxyl groups, and to a lesser extent amino groups, reduce the zeta potential in alkaline and neutral electrolyte solutions by 20 mV, so that the ZP is around −40 mV. In acidic solutions, the complete dissociation of the amino groups takes place and leads to a positive zeta potential, so that the isoelectric point shifts to the right, to a higher pH value. The difference between the functionalization processes can be seen in the results of the zeta potential.

In the thermosol process, similar to the disperse dyeing process, an aqueous suspension of chitosan particles in the submicron range was applied, which was activated with acetic acid immediately before thermosoling (D3). When applied to standard polyester fabric (STD-D3), the change in zeta potential was more pronounced than when applied to hydrolyzed polyester fabric (STD-H-D3). The IEP for STD-D3 is 5.7 and for STD-H-D3 4.4, which could be due to the hydrophobic nature of the chitosan submicron particles. Since the water molecules have no access to the hydrophilic terminal –OH– and –COOH– groups during sorption due to the very high crystallinity and the strong orientation of the polyester macromolecules, the ester chain groups –C=O–O– cannot form an H-bond with water molecules. Therefore, it is possible that the H-bond interactions between chitosan submicron particles and polyester fibers play a minor role, while other forces such as dispersion forces and hydrophobic interactions could play a dominant role due to the pronounced hydrophobicity of both substances.

According to Sinner, the washing process depends on four factors: chemical action, mechanical agitation, temperature, and time, with water being the medium that combines these factors. Water hardness and pH value also play a role [67,68]. In this study, all factors were the same and were carefully selected to obtain an appropriate evaluation of chitosan functionalization. The temperature was set to the maximum allowable temperature for polyester fabrics of 60 °C, the time to 30 min, the pH to 7—neutral, and soft water was used. In terms of chemistry, the non-ionic surfactant Felosan NOF was used instead of a conventional detergent. Felosan NOF has been specially developed for the removal of oil and grease stains that occur on synthetic fibers. Due to its chemical composition based on fatty alcohol ethoxylate, it offers excellent emulsifying properties and good universal washing properties. Study of water systems containing chitosan and non-ionic surfactants has also confirmed that the water systems are slightly affected by the critical micellar concentration of the surfactant, indicating a weak hydrophobic interaction between non-ionic surfactants and chitosan in water [69]. Since all factors of the washing process were consistent and the non-ionic surfactant used does not interact with chitosan, the differences in the results obtained are solely due to the bonds between chitosan and polyester fabric. Looking at the ZP values shown in Figure 2 and Figure 3 after one washing cycle, it can be assumed that the unbound submicron particles of chitosan could be easily removed during the washing process. The ZP results indicate that chitosan was still present in the polyester fabrics, but in much smaller quantities. Therefore, the IEP value was at a lower pH value, confirming the removal of submicron chitosan particles (the IEP value for STD-D3 decreased from 5.7 to 4.2 for STD-D3-1W; and for STD-H-D3, from 4.4 to 4.0 for STD-H-D3-1W). Another reason for the lower adsorption of chitosan submicron particles on activated polyester fabric by accelerated alkali hydrolysis with HDTMAC compared to standard polyester fabric could be the charge. As mentioned above, a small amount of HDTMAC can repel chitosan due to the same cationic charge.

Functionalization with homogenized chitosan gel in the pad-dry-cure process leads to better properties. The zeta potential is similar at a pH of 7.8; for STD-3 ZP, it was −34.95 mV, for STD-H-3 ZP was −33.43 mV, and it did not change after one wash cycle. The achieved IEP for STD-3 was 6.0 and for STD-H-3 5.7, and this changed slightly during the washing process.

**Figure 4:**
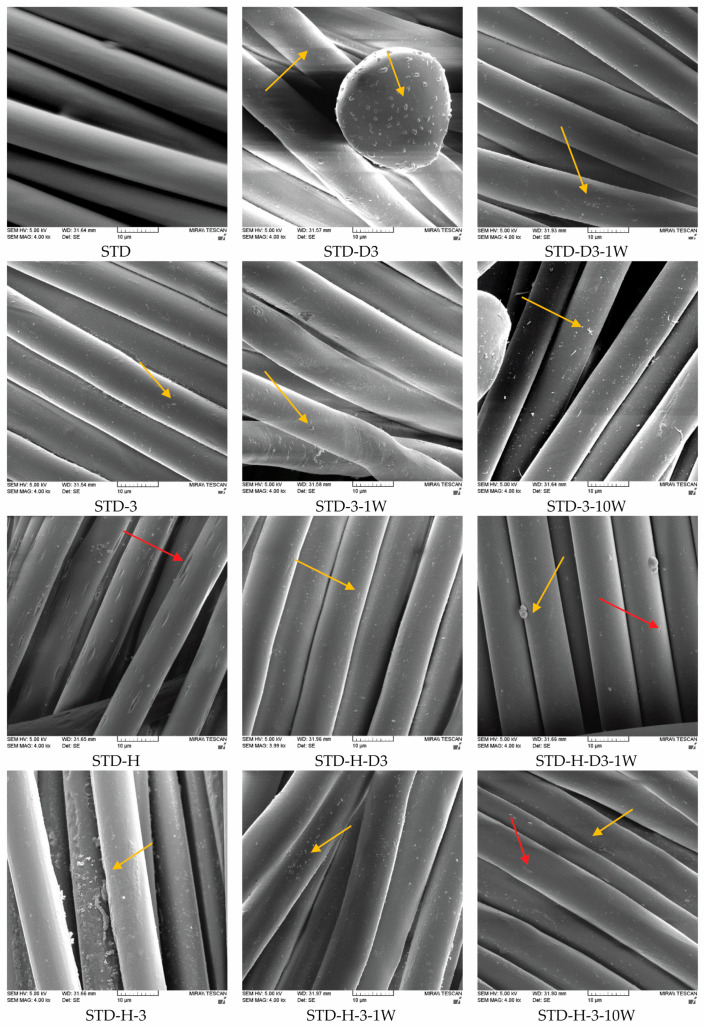
SEM micrographs of standard (STD) and hydrolyzed (STD-H) fibers in polyester fabrics at a magnification of 4000×, after chitosan functionalization (–3), 1 and 10 washing –W, –10W) cycles. Yellow arrows: chitosan, red arrows: pits.

Morphological characterization of the fiber surface was performed to confirm the results of the ZP. Figure 4 shows the micrographs of the polyester fibers in the fabric taken with a scanning electron microscope (SEM) at a magnification of 4000×. It can be seen that the fibers in the standard polyester fabric (STD) have a smooth surface. The application of chitosan leads to the appearance of granular structures on the surface (yellow arrows in Figure 4), indicating the presence of chitosan on the fiber surface. This is particularly visible on the surface of fabrics treated with a dispersion of chitosan submicron particles (STD-D3) using the thermosol process, while the granular structures are much smaller when applied as a homogenized gel (STD-3). After a wash, the amount of particles on the surface of the thermosol-treated fabric (STD-D3-1W) was small, which confirms the ZP results indicating that the chitosan submicron particles were washed off. However, the granular structures on the surface of the polyester fabric treated with homogenized gel are visible after the first wash, confirming the stability of the treatment as shown by the zeta potential measurements.

Alkali hydrolysis leads to characteristic pits (red arrows in Figure 4) on the surface of the fibers [9,13], which are caused by the action of alkali (STD-H). The chitosan filled the pits caused by the alkali and they became less visible or no longer visible at all. The washing process reduced the number of submicron chitosan particles on the fiber, so that the pits on the STD-H-D3-1W fabric became visible again. This may be because additional pilling occurred during the washing process. However, after treatment of the hydrolyzed fabric with homogenized chitosan gel by pad-dry-cure, a large number of particles were visible on the fiber surface (STD-H-3-1W). The chitosan was already present on the fiber after the first wash cycle, which correlates with the zeta potential results.

**Figure 5:**
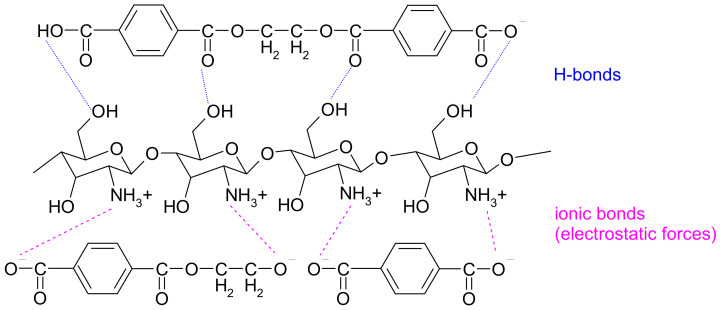
Chitosan bonding to polyester fabrics.

In view of the results presented and the durability of the chitosan functionalization treatment after the first washing cycle with a non-ionic surfactant, functionalization with chitosan homogenized in acetic acid solution by the pad-dry-cure process proved to be more favorable than the thermosol process with dispersion of submicron chitosan particles. It can be assumed that only H-bonds are present in the thermosol process, while ionic bonds also occur between protonated chitosan and polyester fiber in the pad-dry-cure process (Figure 5).

**Figure 6:**
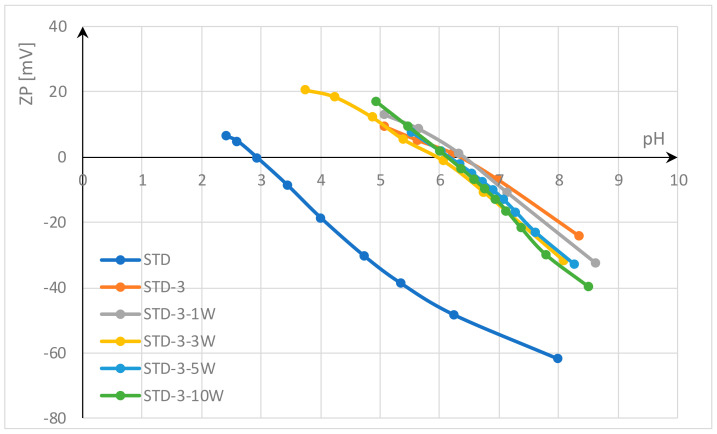
Zeta potential of standard polyester fabrics (STD) functionalized with homogenized chitosan gel (−3) and after 1, 3, 5 and 10 washing cycles.

**Figure 7:**
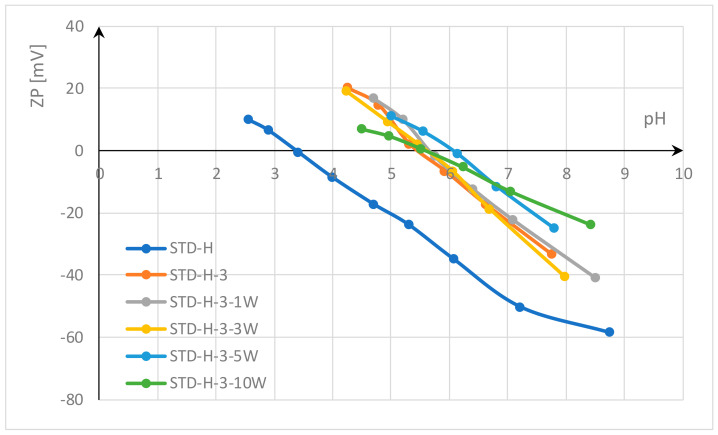
Zeta potential of hydrolyzed polyester fabrics (STD-H) functionalized with homogenized chitosan gel (−3) and after 1, 3, 5 and 10 washing cycles.

Since functionalization with homogenized chitosan gel in the pad-dry-cure process led to better results, such functionalized polyester fabrics were subjected to 10 wash cycles in a solution of the non-ionic surfactant Felosan NOF and water to determine their durability. As already mentioned, the non-ionic surfactant used for washing has no influence on the zeta potential measurements. The zeta potential results of standard and hydrolyzed polyester fabrics functionalized with homogenized chitosan gel after functionalization and after washing shown in Figure 6 and Figure 7 indicate that no significant change in zeta potential occurred during the 10 washing cycles. Although it was expected that the ZP would change due to fiber damage and fibrillation caused by abrasion of the fabric during the washing process [58], this did not occur. It can only be seen that a kind of re-deposition of chitosan took place in the hydrolyzed polyester fabric (STD-H-3), so that the functionalized hydrolyzed polyester fabrics show the highest zeta potential after 5 and 10 washing cycles. SEM micrographs of standard and hydrolyzed fibers in polyester fabrics shown in Figure 4 confirm these results. After 10 washing cycles, particles are still visible on the fiber surface of the fabrics treated with homogenized chitosan gel in the pad-dry-cure process.

Considering the results of the ZP and SEM analyses, it can be said that the functionalization with chitosan homogenized gel by pad-dry-cure process is durable. The mechanical, spectral and antimicrobial properties were then determined.

In order to determine the changes in the mechanical properties of the fabric, the tensile properties and the mass per unit area were determined using standard methods. The tensile properties of polyester fabrics after chitosan functionalization and after 1, 3, 5 and 10 washing cycles, expressed by the breaking force (*F*) and elongation (*ε*), and their changes are shown in Table 2. The mass of the fabric per unit area (*m*) and its change compared to the initial fabric are also given.

Standard polyester fabric (STD) has a mass per unit area of 170 g/m^2^, breaking force value of 1914.50 N and an elongation of 22.65%. As expected, alkali hydrolysis led to a significant decrease in breaking force by 6.89% and elongation by 7.95% due to the de-weighting of fabric (*m* = 158.88 g/m^2^). Looking at the optimum values of weight loss and loss of breaking force after alkali hydrolysis, it can be concluded that the process was well performed. The thermosol process contributes to the heat setting of polyester fabrics, so that the functionalization leads to an even higher strength than before. The results of mass per unit area indicate that shrinkage also occurred, so the higher strength can be attributed to the higher fabric mass. Functionalization with homogenized chitosan gel had no effect on the strength of the fabric, as the change in tensile strength was only 1% and in weight 1.23%. In functionalized hydrolyzed polyester fabrics, the chitosan binds to new active groups on the surface of the polyester fibers and forms a chitosan layer on the fabric surface, increasing the mass per unit area and reducing the strength loss caused by hydrolysis. During the washing process, the fabric may be worn out, but since a neutral non-ionic surfactant was used, the shrinkage of the fabric and the mechanical damage were much less than when using detergents, so that the strength loss after 10 washing cycles was only 4%. This finding confirms the results of mass per unit area.

As treatment with acid can lead to yellowing, the spectral properties were measured. Whiteness, yellowing index, tint value (TV) and tint deviation (TD) with their coloristic meaning were automatically calculated from the spectral remission. The results are shown in Table 3. Standard polyester fabric has a degree of whiteness of 77.9 with no appreciable deviation in tint from the white standard. Alkali hydrolysis cleaned the surface and resulted in a whiter fabric with a degree of whiteness of 79.6. Functionalization with chitosan led to a slight reduction in the degree of whiteness. This phenomenon is more pronounced with thermosol processes and with hydrolyzed fabric. In the thermosol process, the temperature is higher than in the curing process, and the chitosan is present in yellowish submicron particles. It should be noted that hydrolysis results in small pits that scatter the light, and if submicron chitosan particles were applied, the yellowish color would come to the fore. If the functionalization was carried out with chitosan transparent homogenized gel, there would be no significant change in whiteness. In the washing process up to five washing cycles, no change occurs, but there are slight changes due to the influence of mechanical movement. However, these changes are so small that no significant deviation in tint from the white standard can be detected. The reason for this is, as already mentioned, the use of non-ionic surfactant instead of conventional detergents, so that there was no influence on the degree of whiteness by oxidative bleaching agents or FWAs.

As moisture management is one of the most important performance criteria for the comfort of functional fabrics, the liquid moisture management properties were determined in accordance with AATCC TM 195-2017 using the Moisture Management Tester (MMT). The results of the moisture management properties of polyester fabrics before and after alkali hydrolysis, functionalization with homogenized chitosan gel and after 1, 3, 5 and 10 washing cycles are shown in Table 4 as average (A) of the wetting time (WT), the absorption rate (AR), the maximum wetted radius (MWR) and the spreading speed (SS) for the top (T) and bottom (B) surfaces. The Accumulative One-way Transport Capability (R) and Overall (liquid) Moisture Management Capability (OMMC) were calculated, and the type of fabric was determined as Water Penetration Fabric (WPF) or Moisture Management Fabric (MMF).

The wetting time (WT) measured in MMT is the period of time during which the top and bottom surfaces of the fabric just begin to wet, and corresponds to the drop test [70,71]. The results show that it takes 67 s for a standard polyester fabric to start wetting. Functionalization with chitosan reduced the wetting time of the top surface to 53.9 s (STD-3), and when the number of wash cycles was increased, the wetting time was further reduced. The alkaline hydrolyzed polyester fabric (STD-H) is hydrophobic, and the wetting time was 120 s. It is possible that some oligomers are still present on the fiber surface. However, functionalization with chitosan reduced the wetting time to 20.29 s, which is even better than with functionalized standard polyester fabrics. The wetting time decreased with each washing cycle, so that it dropped to 16.18 s after the first wash cycle and to 10.71 s after the tenth wash cycle.

The absorption rate (AR) indicates the average speed of liquid moisture absorption for the top and bottom surfaces of the sample during the initial change in water content during a test. The sorption increased with chitosan functionalization and the number of wash cycles for both the standard fabric and the chitosan-functionalized fabrics. The sorption of the standard polyester fabric (STD) on the top surface increased slowly, i.e., 6.38%/s, while it increased moderately quickly after functionalization with chitosan (STD-3), namely by 14.74%/s. The bottom surface of both fabrics showed a greater increase in absorption before and after functionalization with chitosan than the top surface of the fabric.

Since the alkaline hydrolyzed polyester fabric (STD-H) is hydrophobic, there was no absorption. However, chitosan functionalization led to a higher absorption rate, which is similar or even better than for functionalized standard polyester fabric. Chitosan brings polar binding sites for water molecules, which is the reason for this higher sorption. The spreading speed represents the accumulated rate of surface wetting from the center of the sample into which the test solution is dropped to the maximum wetted radius. It can be seen that chitosan functionalization contributed to a higher maximum wetted radius, but at the same time the spreading speed was slower due to the polar sites.

The accumulative one-way transport capability is the difference between the area of the liquid moisture content curves of the top and bottom surfaces of a sample as a function of time. For a standard polyester fabric, it is 924.82%, which means that the water content on the bottom surface is much higher than on the top surface, indicating low absorbency. Functionalization with chitosan slightly improved the one-way transport to 853.3%, and with each washing cycle the one-way transport improved to 525.29% for STD-3-10W. Alkali hydrolysis resulted in slower one-way transport, 979.32%. However, more chitosan is bound during functionalization and the one-way transport improved to 676.11%. With each washing cycle the one-way transport improved further, and after the 5th washing cycle it was similar to that of a standard cotton fabric [70], suggesting that this fabric has better sorption properties and is therefore more comfortable to wear.

Overall (liquid) moisture management capability (OMMC) is an index of the overall capability of a fabric to transport liquid moisture. It is calculated by combining three measured performance characteristics: the liquid moisture absorption rate on the bottom surface, the one-way liquid transport capability, and the maximum liquid moisture spreading speed on the bottom surface [70]. Standard polyester fabrics, even after chitosan functionalization, are characterized as Water penetration fabrics (WPF) because they have a small or no spreading area, but very good to excellent one-way transport. Alkali hydrolyzed fabric, even if functionalized with chitosan, shows better properties but has the same characterization. However, after five washing cycles, the alkali hydrolyzed fabric functionalized with chitosan (STD-H-3-5W) had a large spreading area and excellent one-way transport and was therefore characterized as Moisture Management Fabric (MMF), which proves that this fabric has improved hydrophilicity.

**Figure 8:**
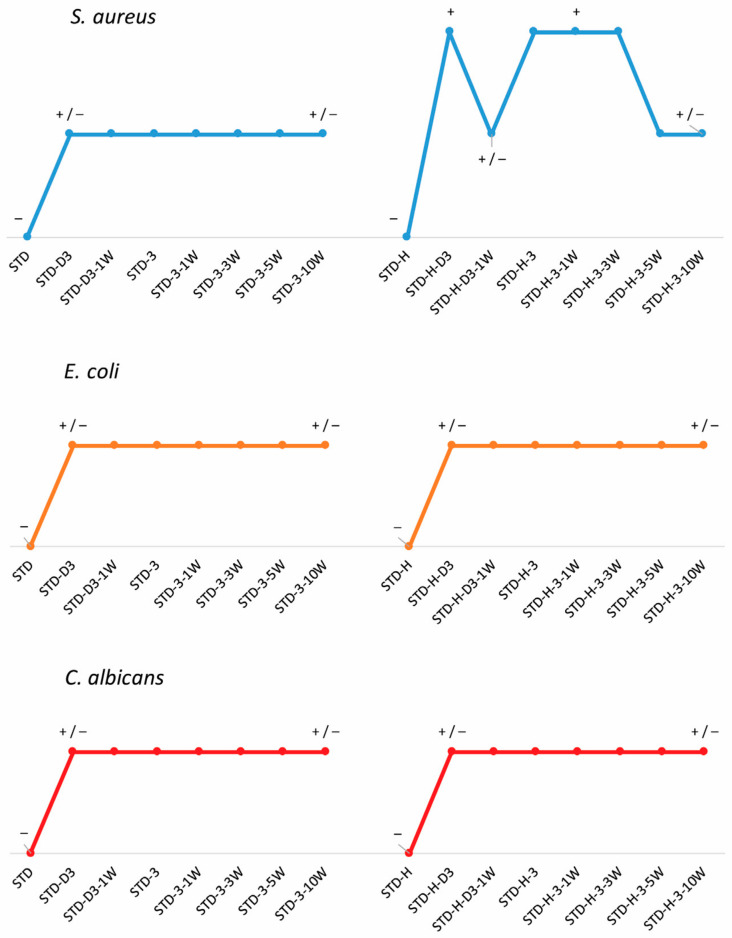
The antimicrobial activity of fabrics before and after functionalization with chitosan and 1, 3, 5 and 10 washing cycles. (+)—antimicrobial activity (inhibition zone can be observed); (+/−)—antimicrobial activity (no colonies beneath); (−)—no antimicrobial activity.

The antimicrobial activity of fabrics before and after functionalization with chitosan and 1 and 10 washing cycles was determined according to AATCC TM 147-2016 against the following microorganisms: the Gram-positive bacterium *Staphylococcus aureus* (*S. aureus*), the Gram-negative bacterium *Escherichia coli* (*E. coli*) and the microfungus *Candida albicans* (*C. albicans*). The results are presented in Figure 8.

The evaluation of antibacterial activity includes the observation of inhibition zones and growth under the sample if present. If the inhibition zone can be observed or if there are no bacterial colonies directly under the sample in the contact area, the material has antibacterial activity. When treated with chitosan, all fabrics showed activity against bacteria. The results show that untreated fabrics, standard fabrics or hydrolyzed polyester fabrics have no activity against the microorganisms tested. However, it can be seen that treatment with chitosan leads to antibacterial activity, regardless of the fabric used. Both fabrics showed good activity against the Gram-negative bacterium *E. coli* and the microfungus *C. albicans*. An even better activity can be observed against the Gram-positive bacterium *S. aureus*, and the difference between the chitosan-functionalized fabrics is clearly visible. When chitosan was applied to standard fabric, there was no zone of inhibition, but when it was applied on activated polyester fabric by accelerated alkali hydrolysis with HDTMAC, there was one, and it persisted for up to three washing cycles. The reason for this could be the HTDMAC used for activation, which also acts as an antimicrobial agent. Since the functionalization with chitosan was performed immediately after hydrolysis and only one rinse cycle was performed in between to remove the oligomers, it is possible that some amount of HDTMAC remained adsorbed on the fabric surface, as suggested by the zeta potential results, and provided additional antimicrobial activity. It should be emphasized that the antimicrobial activity obtained was maintained after 10 washing cycles, indicating a sufficient amount of chitosan in the fabric structure, as suggested by the zeta potential measurement and the SEM images.

## 4. Conclusions

In this work, the functionalization of polyester fabrics with chitosan was investigated by thermosol processes in the form of submicron particles and pad-dry-cure processes with homogenized gel. In the interests of green and sustainable processing, no crosslinking agent was used. The functionalization was carried out on untreated (standard) polyester fabric and on polyester fabric activated by hexadecyltrimethylammonium chloride (HDTMAC)-accelerated alkali hydrolysis. The functionalization of chitosan was evaluated in terms of zeta potential, mechanical (tensile), spectral, moisture management and antimicrobial properties.

A water suspension of chitosan submicron particles was applied by the thermosol method, similar to disperse dyeing, and chitosan was activated with acetic acid after thermosoling. The result obtained was good, but the unbound chitosan particles were easily removed during the first washing cycle. However, functionalization with homogenized chitosan gel using the pad-dry-cure process led to significantly better results and durability.

The functionalization of hydrolyzed polyester fabric with homogenized chitosan gel by the pad-dry-cure process resulted in excellent antimicrobial efficacy and improved mechanical and spectral properties of the fabric, which were maintained even after 10 washing cycles. The zeta potential and SEM images confirm a sufficient amount of chitosan in the fabric. In addition, these fabrics became moisture management fabrics after the 5th washing cycle, indicating excellent wearer comfort.

It should be emphasized once again that chitosan is an environmentally friendly chemical that has no negative impact on humans or the environment when used. It was shown that in the thermosol process, and especially in the pad-dry-cure process, it is possible to incorporate chitosan particles (macromolecules) into the surface layers of polyester fibers. Such a modification has the advantage that no active substances are introduced into the product that could have undesirable biological effects, particularly on the wearer’s skin, which can be the case when crosslinking agents are used.

## Figures and Tables

**Table 1:** Labels and treatments of polyester fabrics.

Label	Treatment
STD	Untreated standard polyester fabric
−H	Alkali hydrolysis
−D3	Functionalization with water suspension of chitosan submicron particles by thermosol process similar to disperse dyeing
−3	Functionalization with homogenized chitosan gel by pad-dry-cure process
−1W, 3W, 5W, 10W	1, 3, 5 or 10 washing cycles

**Table 2:** Tensile properties (breaking force (*F*) and elongation (*ε*)) and mass per unit area (*m*) of polyester fabrics and their changes after chitosan functionalization and after 1, 3, 5 and 10 washing cycles.

**Fabric**	***F*** **[N]**	**CV [%]**	**Δ*F* [%]**	***ε*** **[%]**	**CV [%]**	**Δ*ε* [%]**	***m*** **[g/m^2^]**	**Δ*m***
STD	1914.50	2.05		22.65	2.04		170.00	-
STD-D3	2007.00	2.87	−4.83	23.85	2.18	−5.30	177.72	−4.54
STD-D3-1W	1891.00	1.45	1.23	24.00	3.19	−5.96	171.72	−1.01
STD-3	1890.00	1.32	1.28	23.25	1.38	−2.65	172.09	−1.23
STD-3-1W	1898.50	2.97	0.84	25.20	0.98	−11.26	172.41	−1.42
STD-3-3W	1903.50	3.23	0.57	25.46	0.21	−12.41	172.65	−1.56
STD-3-5W	1860.50	1.77	2.82	25.50	0.36	−12.58	172.47	−1.45
STD-3-10W	1837.00	2.01	4.05	25.50	1.25	−12.58	167.57	1.43
STD-H	1782.50	1.39	6.89	20.85	1.11	7.95	158.88	6.54
STD-H-D3	1762.50	1.45	7.94	21.90	0.06	3.31	160.40	5.65
STD-H-D3-1W	1737.50	1.73	9.25	22.50	1.49	0.66	159.99	5.89
STD-H-3	1854.00	0.90	3.16	24.30	2.08	−7.28	173.42	−2.01
STD-H-3-1W	1783.00	0.74	6.87	24.00	0.90	−5.96	164.70	3.12
STD-H-3-3W	1825.50	1.36	4.65	24.30	2.32	−7.28	162.78	4.25
STD-H-3-5W	1799.50	2.35	6.01	23.67	1.13	−4.48	162.37	4.49
STD-H-3-10W	1774.00	1.47	7.34	23.55	1.25	−3.97	161.11	5.23

**Table 3:** Spectral properties of polyester fabrics—degree of whiteness (*W_CIE_),* yellowing index *(YI)*, tint value (*TV*) and tint deviation (*TD*) with its coloristic meaning after chitosan functionalization and after 1, 3, 5 and 10 washing cycles.

Fabric	*W_CIE_*	*YI*	*TV*	*TD* and Its Coloristic Meaning
STD	77.9	3.0	0	no appreciable deviation in tint from the white standard
STD-D3	76.6	3.6	0	
STD-D3-1W	76.9	3.5	0	
STD-3	77.0	3.5	0.1	
STD-3-1W	75.6	3.8	0.1	
STD-3-3W	77.2	3.4	0.1	
STD-3-5W	77.9	3.3	0.1	
STD-3-10W	76.4	3.6	0.1	
STD-H	79.6	2.6	0.1	
STD-H-D3	73.5	4.2	−0.2	
STD-H-D3-1W	73.7	4.5	−0.1	
STD-H-3	78.2	3.2	0	
STD-H-3-1W	77.1	3.5	0	
STD-H-3-3W	78.1	3.1	0.1	
STD-H-3-5W	78.0	3.1	0.1	
STD-H-3-10W	73.7	4.3	0.1	

**Table 4:** Moisture management properties of polyester fabrics before and after alkali hydrolysis, functionalization with homogenized chitosan gel and after 1, 3, 5 and 10 washing cycles.

		WT (s)		AR (%/s)		MWR (mm)		SS (mm/s)		R (%)	OMMC	Type
		T	B	T	B	T	B	T	B			
STD	A	67.18	3.42	6.38	59.24	5.0	6.25	4.52	1.45	924.82	0.67	WPF
	CV	0.84	0.28	0.84	0.08	0.81	0.4	1.95	0.21	0.12	0.02	
STD-3	A	53.90	5.04	14.74	60.84	5.0	6.25	0.12	1.02	853.30	0.65	WPF
	CV	0.54	0.28	0.93	0.135	0.0	0.4	0.64	0.25	0.14	0.02	
STD-3-1W	A	43.14	4.73	8.99	49.41	8.75	10.0	0.58	1.18	569.38	0.58	WPF
	CV	1.24	0.26	0.75	0.37	0.97	0.70	0.98	0.19	0.51	0.22	
STD-3-3W	A	35.82	10.59	6.32	93.51	7.5	7.5	0.22	0.59	603.39	0.67	WPF
	CV	0.58	0.57	0.31	0.53	0.38	0.38	0.60	0.33	0.31	0.04	
STD-3-5W	A	20.29	7.37	7.52	64.19	5.0	5.0	0.33	0.66	676.11	0.65	WPF
	CV	0.62	0.10	0.27	0.07	0.0	0.0	0.59	0.10	0.13	0.02	
STD-3-10W	A	5.36	5.60	9.97	54.26	13.75	11.25	1.71	1.54	525.29	0.64	WPF
	CV	0.27	0.31	0.50	0.27	0.54	0.56	0.50	0.75	0.31	0.02	
STD-H	A	120.0	4.03	0.0	47.52	0.0	5.0	0.0	1.22	979.32	0.62	WPF
	CV	0.0	0.23	0.0	0.31	0.0	0.0	0.0	0.24	0.03	0.08	
STD-H-3	A	20.29	7.37	7.52	64.19	5.0	5.0	0.33	0.66	676.11	0.65	WPF
	CV	0.62	0.11	0.27	0.072	0.0	0.0	0.59	0.10	0.13	0.01	
STD-H-3-1W	A	16.89	4.41	9.76	61.05	8.75	12.5	5.64	1.52	592.53	0.68	WPF
	CV	1.52	0.41	0.21	0.07	0.28	0.23	1.75	0.68	0.31	0.10	
STD-H-3-3W	A	14.37	4.12	22.55	73.18	15.0	15.0	2.14	2.90	534.17	0.80	WPF
	CV	1.19	0.35	0.33	0.10	0.57	0.57	0.91	0.49	0.34	0.18	
STD-H-3-5W	A	6.68	6.08	10.71	38.00	16.25	16.25	1.62	1.61	431.14	0.61	MMF
	CV	0.33	0.22	0.22	0.30	0.29	0.294	0.33	0.31	0.19	0.04	
STD-H-3-10W	A	10.71	4.85	20.11	30.73	23.75	23.75	1.68	1.78	264.01	0.43	MMF
	CV	1.13	0.08	0.31	0.33	0.20	0.20	0.48	0.31	0.80	0.21	

Average—Mean value (A); Variation coefficient (CV); Wetting Time (WT); Absorption rate (AR); Maximum wetted radius (MWR); Spreading speed (SS) for top (T) and bottom (B) surface; Accumulative One-way Transport Capability (R); Overall (liquid) Moisture Management Capability (OMMC); Water Penetration Fabric (WPF); Moisture Management Fabric (MMF).

## Data Availability

The original contributions presented in this study are included in the article. Further inquiries can be directed to the corresponding author.

## References

[1] Hsieh Y.L., Pastore C., Kiekens P. (2001). Chapter 2: Surface Characteristics of Polyester Fibers. Surface Characteristics of Fibers and Textiles.

[2] Sanders E.M., Zeronian S.H. (1982). An analysis of the moisture-related properties of hydrolyzed polyester. J. Appl. Polym. Sci..

[3] Zeronian S.H., Collins M.J. (1989). Surface Modification of Polyester by Alkaline Treatments. Text. Prog..

[4] Latta B.M. (1984). Improved tactile and sorption properties of polyester fabrics through caustic treatment. Text. Res. J..

[5] Tavanaia H. (2009). A new look at the modification of polyethylene terephthalate by sodium hydroxide. J. Text. Inst..

[6] Grancarić A.M., Soljačić I., Rukavina I., Čavar T. (1988). Utjecaj obrade na efekte alkalne hidrolize poliestera. Tekstil.

[7] Kallay N., Grancarić A.M., Tomić M. (1990). Kinetics of Polyester Fibre Dissolution. Text. Res. J..

[8] Grancarić A.M., Kallay N. (1993). Kinetics of polyester fiber alkaline hydrolysis: Effect of temperature and cationic surfactants. J. Appl. Polym. Sci..

[9] Grancarić A.M., Pušić T., Kallay N. (1991). Modifikacija poliesterskog vlakna alkalnom hidrolizom. Polimeri.

[10] Musale R.M., Shukla S.R. (2017). Weight reduction of polyester fabric using sodium hydroxide solutions with additives cetyltrimethylammonium bromide and [BMIM]Cl. J. Text. Inst..

[11] Gawish S.M., Bourgeois M., Ambroise G. (1986). Effect of Cationic Surfactants on the Alkaline Hydrolysis of Polyester Fabrics. Am. Dyest. Report..

[12] Gawish S.M., Mosleh S., Ramadan A.M. (2002). Synthesis of a new cationic surfactant for the alkaline hydrolysis of solvent-pretreated polyester fabrics. J. Appl. Polym. Sci..

[13] Čorak I., Tarbuk A., Đorđević D., Višić K., Botteri L. (2022). Sustainable Alkaline Hydrolysis of Polyester Fabric at Low Temperature. Materials.

[14] Čorak I., Tarbuk A., Šeparović N., Višić K., Đorđević D., Kirin S., Štedul I., Bubaš M. (2022). Surface modification of polyester fabric at lower temperature. 8th International Professional and Scientific Conference Book of Proceedings, Occupational Safety and Health.

[15] Guebitz G.M., Cavaco-Paulo A. (2008). Enzymes go big: Surface hydrolysis and functionalization of synthetic polymers. Trends Biotechnol..

[16] Čorak I., Tarbuk A., Flinčec Grgac S., Dekanić T. (2024). Bio-Innovative Modification of Poly(Ethylene Terephthalate) Fabric Using Enzymes and Chitosan. Polymers.

[17] Dong Z.Q., Chen G.Q. (2012). Alkaline Hydrolysis of Polyester in the Presence of Ionic Liquids. Adv. Mat. Res..

[18] Cao J., Meng C., Cheng X., Pan X. (2019). Surface alkali deweighting and dyeing of polyester fabric by one-bath and one-step process. Surf. Innov..

[19] Pušić T., Kaurin T., Liplin M., Budimir A., Čurlin M., Grgić K., Sutlović A., Volmajer Valh J. (2023). The Stability of the Chitosan Coating on Polyester Fabric in the washing process. Tekstilec.

[20] Burkinshaw S.M. (2024). The roles of elevated temperature and carriers in the dyeing of polyester fibres using disperse dyes: Part 1 fundamental aspects. Color. Technol..

[21] Braun H. (1983). Particle Size and Solubility of Disperse Dyes. Rev. Prog. Color. Relat. Top..

[22] Tarbuk A., Grancarić A., Jančijev I., Sharma S. (2006). Protection against UV radiation using a modified polyester fabri. Tekstil.

[23] Grancarić A.M., Tarbuk A. (2009). EDA Modified PET Fabric Treated with Activated Natural Zeolite Nanoparticles. Mater. Technol. Adv. Perform. Mater..

[24] Grancarić A.M., Tarbuk A., McCall D. (2007). Surface Modification of Polyester Fabric with Tribomechanically Activated Natural Zeolite (TMAZ) Nanoparticles. Polimeri.

[25] Bakshi P.S., Selvakumar D., Kadirvelu K., Kumar N.S. (2020). Chitosan as an environment friendly biomaterial—A review on recent modifications and applications. Int. J. Biol. Macromol..

[26] Shahid-ul-Islam, Butola B.S. (2019). Recent advances in chitosan polysaccharide and its derivatives in antimicrobial modification of textile materials. Int. J. Biol. Macromol..

[27] Shabbir M., Rather L.J., Mohammad F., Ahmed S., Ikram S. (2017). Chitosan: Sustainable and Environmental-Friendly Resource for Textile Industry. Chitosan: Derivatives, Composites and Applications.

[28] Fras Zemljič L., Bračič M., Ristić T., Šauperl O., Strnad S., Peršin Z., Vasile C. (2019). Functionalization of polymer materials for medical applications using chitosan nanolayers. Polymeric Nanomaterials in Nanotherapeutics.

[29] Çaykara T., Sande M.G., Azoia N., Rodrigues L.R., Silva C.J. (2020). Exploring the potential of polyethylene terephthalate in the design of antibacterial surfaces. Med. Microbiol. Immunol..

[30] Simončič B., Tomšič B. (2010). Structures of Novel Antimicrobial Agents for Textiles—A Review. Text. Res. J..

[31] Flinčec Grgac S., Tarbuk A., Dekanić T., Sujka W., Draczyński Z. (2020). The Chitosan Implementation into Cotton and Polyester/Cotton Blend Fabrics. Materials.

[32] Flinčec Grgac S., Biruš T.-D., Tarbuk A., Dekanić T., Palčić A. (2023). The Durable Chitosan Functionalization of Cellulosic Fabrics. Polymers.

[33] Sunder E., Nalankilli G. (2014). Croslinking of Chitosan with Cotton using Polycarboxylic Acids. Int. J. Eng. Res. Technol..

[34] Chung Y.-S., Lee K.-K., Kim J.-W. (1998). Durable Press and Antimicrobial Finishing of Cotton Fabrics with a Citric Acid and Chitosan Treatment. Text. Res. J..

[35] Latańska I., Kolesińska B., Draczyński Z., Sujka W. (2020). The use of chitin and chitosan in manufacturing dressing materials. Prog. Chem. Appl. Chitin Its Deriv..

[36] Tang W., Wang J., Hou H., Li Y., Wang J., Fu J., Lu L., Gao D., Liu Z., Zhao F. (2023). Review: Application of chitosan and its derivatives in medical materials. Int. J. Biol. Macromol..

[37] Kim J.H., Lee S.Y. (2014). Surface Modification of PET Film and Fabric by Oligo-Chitosan Treatment. Fibers Polym..

[38] Hosseinnejad M., Jafari S.M. (2016). Evaluation of different factors affecting antimicrobial properties of chitosan. Int. J. Biol. Macromol..

[39] Furuike T., Komoto D., Hashimoto H., Tamura H. (2017). Preparation of chitosan hydrogel and its solubility in organic acids. Int. J. Biol. Macromol..

[40] Sikorski D., Gzyra-Jagiela K., Draczynski Z. (2021). The Kinetics of Chitosan Degradation in Organic Acid Solutions. Mar. Drugs.

[41] Sikorski D., Bauer M., Frączyk J., Draczyński Z. (2022). Antibacterial and Antifungal Properties of Modified Chitosan Nonwovens. Polymers.

[42] Choi B.K., Kim K.Y., Yoo Y.J., Oh S.J., Choi J.H., Kim C.Y. (2001). In vitro antimicrobial activity of a chitooligosaccharide mixture against Actinobacillus actinomycetemcomitans and Streptococcus mutans. Int. J. Antimicrob. Agents.

[43] Rabea E.I., Badawy M.E.T., Stevens C.V., Smagghe G., Steurbaut W. (2003). Chitosan as antimicrobial agent: Applications and mode of action. Biomacromolecules.

[44] Ke C.L., Deng F.S., Chuang C.Y., Lin C.H. (2021). Antimicrobial actions and applications of chitosan. Polymers.

[45] Hassan M.I., Mohamed A.F., Taher F.A., Kamel M.R. (2016). Antimicrobial activities of chitosan nanoparticles prepared from Lucila Cuprina Maggots (Diptera Calliphoridae). J. Egypt. Soc. Parasitol..

[46] Hassan M.I., Mohamed A.F., Taher F.A., Kamel M.R. (2016). Chitosan nanoparticles prepared from Lucilia Cuprina Maggots as antibacterial agent. J. Egypt. Soc. Parasitol..

[47] Hassan M., El-dek S., Mohamed A., Abdelwahab A. (2019). In Vitro Assessment of Antimicrobial Activity of Chitosan Nanoparticles Loaded with the Honeybee, Apis mellifera Venom. Egypt. Acad. J. Biol. Sciences. A Entomol..

[48] Zhang W., Zhang J., Xia W. (2014). Effect of Ball-Milling Treatment on Physicochemical and Structural Properties of Chitosan. Int. J. Food Prop..

[49] Czechowska-Biskup R., Wach R.A., Rosiak J.M., Ulański P. (2018). Procedure for determination of the molecular weight of chitosan by viscometry. Prog. Chem. Appl. Chitin Its Deriv..

[50] Qian J., Wang X., Chen Y., Mo C., Liang C., Guo H. (2023). The correlation of molecule weight of chitosan oligomers with the corresponding viscosity and antibacterial activity. Carbohydr. Res..

[51] Li J., Fu J., Tian X., Hua T., Poon T., Koo M., Chan W. (2022). Characteristics of chitosan fiber and their effects towards improvement of antibacterial activity. Carbohydr. Polym..

[52] Gomes L.C., Faria S.I., Valcarcel J., Vázquez J.A., Cerqueira M.A., Pastrana L., Bourbon A.I., Mergulhão F.J. (2021). The Effect of Molecular Weight on the Antimicrobial Activity of Chitosan from Loligo opalescens for Food Packaging Applications. Mar. Drugs.

[53] van der Wal A., Norde W., Zehnder A.J.B., Lyklema J. (1997). Determination of the total charge in the cell walls of Gram-positive bacteria. Colloids Surf. B Biointerfaces.

[54] Cremin K., Jones B.A., Teahan J., Meloni G.N., Perry D., Zerfass C., Asally M., Soyer S., Unwin P.R. (2020). Scanning Ion Conductance Microscopy Reveals Differences in the Ionic Environments of Gram-Positive and Negative Bacteria. Anal. Chem..

[55] UN Environment Programme: Green and Sustainable Chemistry. https://www.unep.org/topics/chemicals-and-pollution-action/circularity-sectors/green-and-sustainable-chemistry.

[56] (2016). Surface Active Agents—Evaluation of Certain Effects of Laundering—Methods of Preparation and Use of Unsoiled Cotton Control Cloth.

[57] Grancarić A.M., Tarbuk A., Pušić T. (2005). Electrokinetic properties of textile fabrics. Color. Technol..

[58] Luxbacher T., Pušić T., Bukšek H., Petrinić I. (2016). The zeta potential of textile fabrics: A review. Tekstil.

[59] (1977). Textiles—Woven Fabrics—Determination of Mass per Unit Length and Mass per Unit Area.

[60] (2013). Textiles—Tensile Properties of Fabrics—Part 1: Determination of Maximum Force and Elongation at Maximum Force Using the Strip Method.

[61] (1997). Textiles—Tests for Color Fastness—Part J02: Instrumental Assessment of Relative Whiteness.

[62] Griesser R. (1994). Assessment of whiteness and tint of fluorescent substrates with good inter-instrument correlation. Color Res. Appl..

[63] (1980). Description of Yellowness of Near-White or Near-Colourless Materials.

[64] (2017). Liquid Moisture Management Properties of Textile Fabrics.

[65] (2017). Antibacterial Activity of Textile Materials: Parallel Streak.

[66] Pušić T., Grancarić A.M., Tarbuk A., Šauperl O., Soljačić I. (2010). Adsorption and Desorption of Ionic Surfactants. Tenside Surfactants Deterg..

[67] Jakobi G. (1987). Detergents and Textile Washing.

[68] Smulders E. (2002). Laundry Detergents.

[69] Pepić I., Filipović-Grčić J., Jalšenjak I. (2009). Bulk properties of nonionic surfactant and chitosan mixtures. Colloids Surf. A Physicochem. Eng. Asp..

[70] Dekanić T., Tarbuk A., Flinčec Grgac S. (2018). The liquid moisture management properties of low-temperature cured water-repellent cotton fabrics. Tekstil.

[71] Tarbuk A., Flinčec Grgac S., Dekanić T. (2019). Wetting and Wicking of Hospital Protective Textiles. Adv. Technol..

